# Cortical Excitability Before and After Long‐Term Perampanel Treatment for Epilepsy

**DOI:** 10.1002/acn3.70044

**Published:** 2025-04-17

**Authors:** Robert M. Helling, Johannes P. van Dijk, Prisca R. Bauer, Roland D. Thijs, Josemir W. Sander, Machiel Zwarts, Gerhard H. Visser

**Affiliations:** ^1^ Stichting Epilepsie Instellingen Nederland (SEIN) Heemstede the Netherlands; ^2^ Academic Center of Epileptology Kempenhaeghe Heeze the Netherlands; ^3^ Faculty of Electrical Engineering Eindhoven University of Technology Eindhoven the Netherlands; ^4^ Department of Orthodontics Ulm University Ulm Germany; ^5^ Institute of Musicians’ Medicine University Medical Center Freiburg Freiburg im Breisgau Germany; ^6^ Department of Neurology Leiden University Medical Centre Leiden the Netherlands; ^7^ UCL Queen Square Institute of Neurology London UK; ^8^ Chalfond Centre for Epilepsy Chalfont St Peter UK; ^9^ Department of Neurology West China Hospital Chengdu China

**Keywords:** AMPA‐receptor antagonist, intracortical facilitation, resting motor threshold, TMS‐evoked EEG potential, transcranial magnetic stimulation

## Abstract

**Objective:**

Antiseizure medications (ASMs), which may influence cortical excitability, are the mainstay of epilepsy treatment. Transcranial magnetic stimulation (TMS) helps evaluate cortical excitability. We assessed changes in TMS responses using serial TMS measurements in people treated with an adjunctive noncompetitive AMPA‐receptor antagonist.

**Methods:**

We included adults with refractory, active epilepsy (≥ 1 seizure/month), advised to start adjunctive treatment with the noncompetitive AMPA‐receptor antagonist perampanel as outpatients. After informed consent, we performed TMS measurement at three points: baseline before starting perampanel, at around 2 months after starting (4 mg/day), and at a final/effective dose around 6 months. Dependent on seizure reduction (> 50%), participants were dichotomized into responders (Rs) and nonresponders (NRs). We compared changes in motor cortex excitability through the rMT using a linear mixed‐effects model. We evaluated TMS‐evoked potentials (TEPs) to single pulse and paired pulse using within‐subject Monte Carlo–based permutation analysis.

**Results:**

We included 18 adults, of whom 17 (6 R, 11 NR, 1 lost to follow‐up) had baseline and second‐month measurements, and nine (4 R, 5 NR) had all three. In responders, motor cortex excitability, quantified by rMT, significantly increased with increasing dose. Conversely, no significant changes were seen in the NR subgroup. TEPs for the single pulse and paired pulse showed no significant clusters for any peaks between measurement and group comparisons.

**Interpretation:**

The TEPs showed no significant changes between measurements and/or groups. Motor cortex excitability quantified by rMT is a potential biomarker to track or predict treatment outcomes in people starting adjunctive perampanel for epilepsy.

## 1 Introduction

The normal functioning of cortical networks critically depends on a finely tuned level of excitability, believed to be a product of excitation and inhibition within networks [1, 2]. Monitoring cortical excitability in brain networks is advantageous for understanding normal and pathological brain function [3, 4]. There are still gaps in understanding fluctuations in cortical excitability [5]. Epilepsy is a condition in which regular brain activity is interrupted by periods of abnormal hypersynchronous activity, that is, seizures. Networks with a shifted or aberrant excitation:inhibition (E:I) balance are thought to facilitate seizures [1, 6]. Treatment with antiseizure medication (ASM) may help restore the E:I balance [7].

Glutamate is the primary excitatory neurotransmitter, acting on ionotropic and metabotropic receptors at the synapse. When glutamate binds to the ionotropic α‐amino‐3‐hydroxyl‐5‐methyl‐4‐isoxazole‐propionate (AMPA) receptor, the receptor gate opens, enabling the transfer of cations and the generation of fast excitatory postsynaptic potentials [8]. The fast synaptic transmission mediated by AMPA receptors allows for synchronization of the firing of pyramidal neurons and can play an essential role in seizure initiation and the spread of seizures [9]. Perampanel is the first ASM that targets the AMPA receptor by blocking the receptor using allosteric regulation, exerting a broadband antiseizure effect [10, 11, 12].

Transcranial magnetic stimulation (TMS) allows for direct noninvasive stimulation of cortical areas [13]. Motor cortex excitability is assessed by stimulating the motor system and recording the motor response in the target muscle. The resting motor threshold (rMT) is the stimulus strength needed to elicit a motor response of sufficient voltage recorded through the electromyogram (EMG). Previous studies using serial TMS measurements within subjects have demonstrated that the rMT can monitor changes in motor cortex excitability when starting a ketogenic diet or tapering medication [14, 15]. Measuring the cortical response through combined TMS‐EEG is a relatively new modality of functional brain mapping [7]. The TMS‐evoked potential (TEP) is a time‐varying signal with multiple peaks at different latencies and locations [16]. Multichannel EEG recordings of cortical responses to TMS have been increasingly used to assess drug effects [17, 18, 19]. A recent pharmaco‐TMS‐EEG study has shown that GABAergic and glutamatergic pharmaceutical agents modulate the TEP shortly after using a single oral dose in healthy subjects [20]. It demonstrated modulation of the peak at 60 ms by perampanel in the nonstimulated hemisphere, suggestive of a role of AMPA receptors in the interhemispheric spread of activity. Previous single‐dose TMS‐EMG studies utilizing paired‐pulse TMS (ppTMS) protocols demonstrated that the NMDA antagonist dextromethorphan and the AMPA‐type glutamate receptor antagonist memantine decreased intracortical facilitation (ICF) while enhancing short‐interval intracortical inhibition (SICI) [21, 22]. Thus, ICF is mainly associated with glutamate receptor‐mediated excitatory functions in the motor cortex. Together, TMS combined with EMG and EEG allows for a multimodal approach for quantifying cortical excitability.

We assessed the effect of long‐term adjunctive perampanel treatment on cortical excitability in people with refractory epilepsy. We conducted a within‐subject controlled longitudinal study to elucidate the effect of long‐term perampanel treatment on cortical excitability measured by TMS‐EEG for single‐pulse TMS (spTMS) and the ppTMS ICF protocols. We compared pretreatment evoked responses to responses at a fixed ASM dose in all subjects to evaluate the effect on motor cortex excitability as measured by the rMT and TEPs. We also compared these measures in Rs and NRs to assess if there are predictive markers and/or diagnostic markers for treatment effect.

## 2 Methods

### 2.1 Population

Adults with refractory localization‐related epilepsy or generalized tonic–clonic seizures with a minimum of one seizure/month advised to start perampanel treatment at specialist epilepsy clinics in Stichting Epilepsie Instellingen Nederland (SEIN) and Academic Center of Epileptology Kempenhaeghe (ACE) were candidates for the study. They were screened for contraindications to TMS other than seizures. Exclusion criteria included deep brain stimulators in situ, pacemakers or other implanted devices other than nervus vagus stimulators, clinical or radiological evidence of major structural abnormalities of the motor cortex or pyramidal tract, pregnancy, evidence of a major neurological or psychiatric condition other than epilepsy, and/or a change in concomitant medication known to affect cortical excitability. All participants provided informed written consent. The ethics committee of Leiden University Medical Center approved the study (CME Leiden, NL53005.058.15).

### 2.2 Experimental Design

TMS measurements were performed before starting titration (T0), a second measurement at a fixed perampanel dose of 4 mg/day (T1), and a final measurement after reaching the maximum tolerated or effective stable dose (T2). Participants kept a seizure diary prospectively for 4 weeks before T0. If the information on seizure frequency was already available, the baseline T0 measurement was scheduled as soon as possible. Perampanel titration followed standard clinical practice and was at the discretion of the treating neurologist. Participants were asked not to smoke, or take alcohol or coffee in the 12 h preceding measurements and maintain a regular sleep pattern the night before the measurement, which was conducted either at 9.00 a.m. or 4.00 p.m. (fixed for each participant) and spread evenly between a.m. and p.m.

### 2.3 Measurement Setup

We used a MagPro X100 magnetic stimulator (Magventure, Farum, Denmark) and a 14 cm diameter parabolic circular coil (type MMC‐140) or a sham TMS coil (type MCF‐P‐B65) in SEIN. In Kempenhaeghe, the Magstim BiStim stimulator (Magstim Co Ltd., Whitland, UK) was used in combination with a 9 cm round coil. Sham stimulation in Kempenhaeghe was performed by rotating the coil 90° along the vertical axis. The round coil was used because it diffusely activates the cortex compared to focal figure‐of‐eight coils and is less sensitive to small changes in coil position [23]. The current direction through the coil influences the direction of the induced magnetic field, resulting in preferential activation of either the left and/or right motor cortex. Muscle activity of the abductor pollicis brevis muscle (belly‐tendon montage) was monitored using a Viking Nicolet EMG system recording at 16 kHz. Electrode positioning was determined as the site that produced the highest MEPs with above‐threshold peripheral stimulation of the median nerve. Both centers recorded EEG using a 64‐electrode EEG amplifier (SEIN: ANT‐EEGO amplifier sampling at 4 kHz, ANT Neuro b.v., Hengelo, the Netherlands; Kempenhaeghe: tMSI Refa amplifier sampling at 2048 Hz, Twente Medical Systems International B.V, Oldenzaal, the Netherlands), in combination with a 64‐channel TMS‐compatible EEG CAP (WaveguardTM cap, ANT Neuro b.V., Hengelo, the Netherlands). Participants were seated in a comfortable chair with their eyes open and arms in the supine position and instructed to blink 1 to 2 s after receiving a TMS stimulus. Earplugs were used to reduce the effect of the auditory‐evoked potential.

### 2.4 Measurement TMS Protocol

TMS stimulation sessions involved a round coil and sham TMS centered above the vertex (Cz electrode position). The rMT was first estimated starting at 30% maximum stimulator output (MSO) with 5% stepwise increments until a motor‐evoked response of > 50 μV in the hand contralateral to the stimulated hemisphere was observed in > 5 out of 10 trials [24]. Next, 1% adjustments were made until the lowest intensity was reached, where > 5 out of 10 trials were above 50 μV. This was repeated for both current directions, which either preferentially activated the right or left hemisphere, depending on the current direction. Then, a session of spTMS (50 pulses at 90% rMT), sham TMS (50 pulses at 90% rMT), and ppTMS ICF (50 pulses, 90% rMT conditioning, 110% rMT test stimulus with 10 ms interstimulus interval) was performed in normal and reversed current directions with 5 s in between stimuli with 20% jitter. The current direction through the round coil was shifted in a cyclical fashion. Between rounds of stimulation, 5‐min breaks were scheduled as a slight pause to instruct and prepare the participant for the session.

### 2.5 Data Processing

The raw EEG data were processed using a combination of in‐house scripts programmed in Matlab (The Math Works Inc. MATLAB, Version 2021a) and the Fieldtrip toolbox for EEG/MEG analysis [25]. Firstly, data were visually inspected to remove artifactual channels and then re‐referenced to the common reference of all remaining EEG channels. Epochs of 2 s were used for each TMS protocol, with 1000 ms pre‐ and 1000 ms poststimulus. Trials that included blink artifacts −100 ms pre‐ to 500 ms poststimulus were removed from the dataset (2.6 ± 1.3 trials on average). The TMS pulse artifact was segmented out from −1 ms pre‐ to 10 ms poststimulus. The mean was subtracted for each trial based on a baseline window of −200 to −50 ms relative to the magnetic stimulus. Data were cleaned using two rounds of independent component analysis (FastICA). The first round was performed over the whole epoch to capture and reject blink, saccade, line noise, and slow decay components. The epoch was redefined to −500 ms pre‐ to 750 ms poststimulus for a subsequent second round of ICA decomposition focused on the TMS‐related pre‐ and poststimulus activation patterns where remaining line noise and time‐locked and continuous muscle components were rejected. Then, we applied a fourth‐order bandpass Butterworth zero‐phase filter from 1 to 80 Hz. A final step of automated quality control was the rejection of trials with a norm covariance of > 2 standard deviations from the dataset (three trials, on average, were removed).

### 2.6 Statistics

For the R/NR group comparison, participants were assigned to the R (> 50% reduction) or NR (≤ 50% reduction) groups depending on seizure frequency at last follow‐up compared to the seizure frequency at baseline.

Magnetic or sham stimulation responses were compared between measurement T0 and T1 for the individual peak time of interest (TOI) windows all located between 15 ms before to 262 ms after the magnetic pulse. For each peak, a predefined window was used to compare groups (P25: 16–34 ms, N45: 38–55 ms, P70: 56–82 ms, N100: 89–133 ms, P180: 173–262 ms) [18]. TEPs were compared for the whole epoch from 15 to 262 ms and the predefined peaks, between measurements, using dependent sample *t*‐tests, or between centers and between Rs/NRs using independent *t*‐tests. Exact *p*‐values were calculated by enumeration using cluster‐based permutation testing to correct for multiple comparisons and the small sample size [26]. Clusters based on adjacency in time and electrode space (minimum of 2 electrodes) were formed using samples with a cluster‐alpha of 0.05 (independent *t*‐test). Each cluster's *t*‐values (for time samples and electrodes) were summed and compared to a dataset of 2500 random permutations of the original data. Clusters were considered significantly different between groups when their summed t‐values were lower or higher than 2.5% (*p* < 0.025) of all permuted clusters.

The rMT was modeled using linear mixed‐effect analysis of the relationship between rMT and perampanel dose, with center (SEIN or Kempenhaeghe) and current direction (preferentially activated hemisphere) as fixed effects. As random effects, we incorporated intercepts for subjects and by‐outcome random slopes for the effect of medication dose. The following formula describes the whole model:

$$\begin{array}{c} \mathrm{rMT}\sim 1+\text{Center}+\text{Current Direction}+\left(1|\text{subject}\right)\\ \;+\left(\text{dose}|\text{response}\right) \end{array}$$

Visual inspection of residual plots did not suggest any apparent deviations from homoscedasticity or normality.

## 3 Results

### 3.1 Participants

Eighteen adults with refractory epilepsy were recruited from SEIN (*n* = 8) and ACE Kempenhaeghe (*n* = 10). Demographics are provided in Table 1, and a flow diagram is shown in Figure S1. All participants well tolerated the TMS measurement. All underwent T0. Seventeen had T1 (one lost to follow‐up), of whom nine had T2. Eight participants dropped out after T1 due to increased dizziness (*n* = 2), fatigue (*n* = 2), emotional state (*n* = 1), nausea (*n* = 1), instability (*n* = 1), or inability to do a specific activity and a feeling that the seizures became more severe (*n* = 1).

**TABLE 1:** Population demographics.

Case	Age (years)	Gender	Handedness	Seizure type	Onset (age)	ASM (*n*)	Seizures (events/month)			Dose (mg)	
							T0	T1	T2	T1	T2
401	47	F	R	FIA	**33**	1	9	7	8	4	10
402	57	M	R	FIA/fbTC	4	2	5	4	4	4	8
403	60	M	R	FIA	55	1	4	0	1	4	8
404	41	M	R	FIA	4	1	8	7	—	4	—
405	64	F	R	FIA	10	3	6	2	—	4	—
406	76	M	R	FIA/fbTC	17	2	3	1	—	4	—
407	59	M	R	FIA/fbTC	10	3	2	2	—	4	—
408	22	F	R	FIA/fbTC	8	1	8	6	—	4	—
409	20	F	L	FIA/fbTC	6	1	10	13	—	4	—
410	19	M	R	FIA	7	1	2	0	0	4	6
411	24	M	R	FIA/fbTC	7	2	1	1	—	4	—
412	51	M	R	FIA	34	2	1	1	—	4	—
413	28	M	R	FIA/fbTC	18	2	4	3	5	4	10
414	29	M	L	FIA/fbTC	30	2	2	2	3	4	8
415	32	M	R	FIA/fbTC	17	2	1	0	0	4	8
416	26	M	R	FIA/fbTC	18	2	3	1	1	4	6
417	44	F	R	FIA/fbTC	40	3	7	—	—	—	—
418	26	F	R	FA/fbTC	21	2	1	1	1	4	6

Abbreviations: fbTC, focal to bilateral tonic–clonic; FIA, focal with impaired awareness; T0, baseline preadjunctive treatment; T1, 4 mg‐dose measurement; T2, max effective/tolerable dose measurement.

In most participants, perampanel was increased to a 4 mg/day dose at a median of 4 weeks. Measurement T1 was performed at a median of 7 weeks. One subject, however, reached the 4 mg dose within 2 weeks of starting treatment, with T1 measured at the end of the fourth week. Measurement T2 was performed on a 6 mg dose in three, 8 mg in four, and 10 mg in two.

The average seizure frequency was 4.15 ± 2.98 events/month at T0, 3.05 ± 3.33 events/month at T1, and 2.50 ± 2.57 events/month at T2. Six subjects responded to adjunctive drug treatment (> 50% seizure reduction compared to baseline) at measurement T1. Two Rs stopped after T1 due to side effects.

### 3.2 Resting Motor Threshold

**FIGURE 1:**
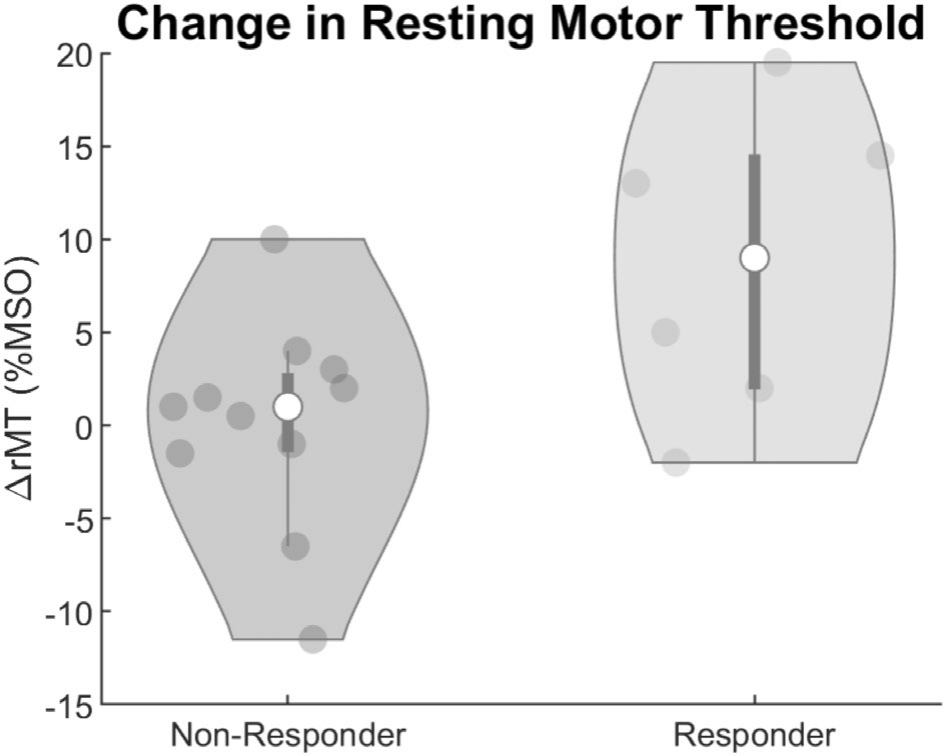
Violin plots of the change in resting motor threshold (rMT) dichotomized by response to adjunctive perampanel administration. They depict the change (∆) in the rMT averaged across hemispheres measured at the 4 mg‐dose measurement (T1) relative to the baseline preadjunctive treatment measurement (T0). The gray dots represent the individual measurements, the white circle represents the mean value, the dark gray bars represent the interquartile range, and the gray area represents the smoothed probability density. MSO, mean stimulator output; rMT, resting motor threshold.

The rMT measured at each center for each measurement session is shown in Table 2. The results of the linear mixed‐effect model are shown in Table 3. There were marked differences in rMT between Rs and NRs, shown in Figure 1. The rMT was significantly increased when increasing medication dose in the R subgroup (estimate: −1.4022%MSO/mg, *p*‐value: < 0.001, CI^−^: −0.82813, CI+: 1.9763), but not in the NR subgroup (estimate: 0.1201, *p*‐value: 0.591, CI−: −0.322, CI+: 0.563). The fixed effect for the center was highly significant, with thresholds measured at SEIN requiring significantly lower stimulator output compared to Kempenhaeghe (estimate: −15.238%MSO/mg, *p*‐value: 0.001, CI−: −24.648, CI+: −5.8275). No significant effect of the stimulated hemisphere was observed.

**TABLE 2:** Overview of changes in resting motor threshold.

Center	Measurement	Cases (*n*)	Resting motor threshold	
			Right hemisphere	Left hemisphere
			%MSO (std)	%MSO (std)
ACE Kempenhaeghe	T0	10	64.7 (11.6)	65.2 (10.6)
	T1	10	68.9 (13.4)	66.3 (12.4)
	T2	4	71 (6.6)	69.5 (9.7)
SEIN	T0	8	49.3 (8.6)	51.6 (8.3)
	T1	7	52.1 (12.7)	52.71 (11.0)
	T2	5	54.4 (15.8)	53.6 (9.7)

Abbreviations: T0, baseline preadjunctive treatment; T2, max effective/tolerable dose measurement; %MSO, percentage of max stimulator output; std, standard deviation; T1, 4 mg‐dose measurement.

**TABLE 3:** Resting motor threshold linear mixed‐effects model.

Parameter name	Estimate (%MSO)	Lower‐95 (%MSO)	Upper‐95 (%MSO)	*p*
**Fixed effects**				
Intercept	64.35%	58.16%	70.54%	< 0.001
Center	−15.17%	−24.57%	−5.78%	0.002
Handedness	0.91%	−1.07%	2.89%	0.364
**Random effects**				
Intercept|Subject	9.35%	6.56%	13.34%	18 levels
Dose|Responders	1.41%/mg	0.84%/mg	1.97%/mg	< 0.001
Dose|Nonresponders	0.15%/mg	−0.25%/mg	0.55%/mg	0.452

Abbreviation: %MSO, percentage of max stimulator output.

### 3.3 TMS‐EEG

Montecarlo cluster statistics for both spTMS and ppTMS protocols for the prespecified TOIs are shown for the baseline T0 current direction comparison (Table S1), the 4 mg perampanel dose T1 versus baseline T0 comparison (Table S2), and the R versus NR subgroup comparison (Table S3). No significant clusters were observed.

#### 3.3.1 Single Pulse

**FIGURE 2:**
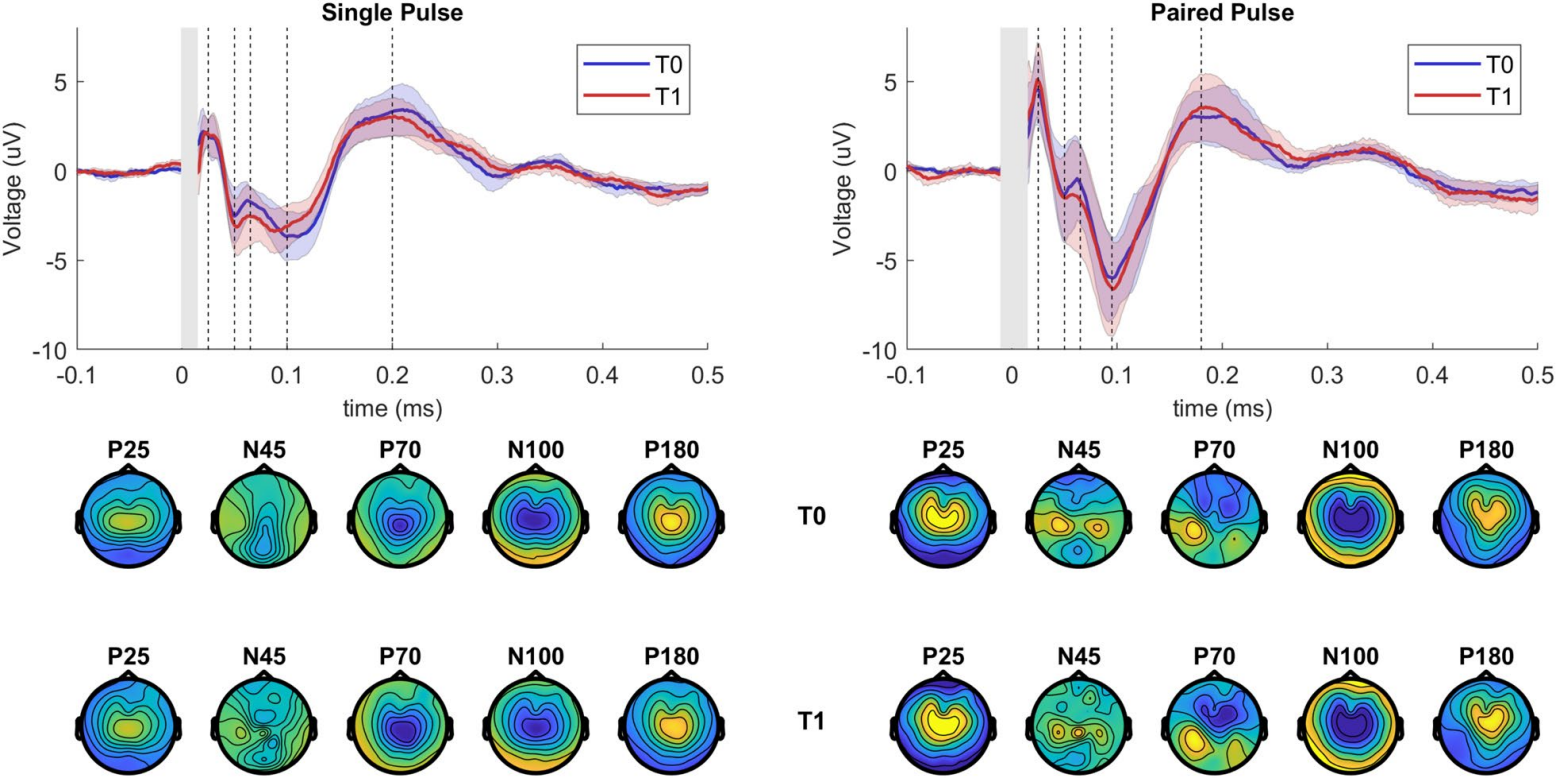
Group average of TEPs evoked by single‐ and paired‐pulse TMS of the left hemisphere before and at 4 mg perampanel dose. Upper panel shows pretreatment baseline (T0) and fixed 4 mg perampanel dose (T1) TEPs averaged across all subjects for the central electrode cluster (Cz and neighboring electrodes with distance < 2). The gray area reflects the segmented window impacted by the TMS pulse artifact. The dotted lines indicate the location of the TEP peaks (P25, N45, P70, N100, and P180). The bottom panels show pretreatment baseline T0 and T1 topographic distributions of the peaks averaged across subjects. Each topography was obtained by averaging the signal in the respective TOI (P25:15–30 ms, N45: 31–55 ms, P70: 56–70 ms, N100: 71–135 ms, P180: 136–250 ms).

**FIGURE 3:**
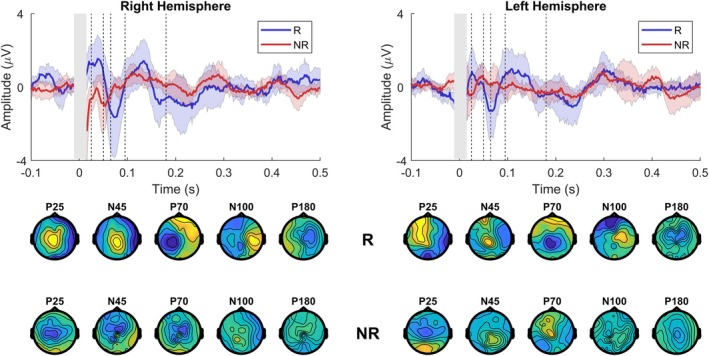
Different waveforms between 4 mg fixed‐dose measurement T1 and baseline T0 TEPs averaged across responders and nonresponders for right hemisphere (left panels) and left hemisphere (right panels) stimulation. Upper panel shows the difference curves between T1 and T0 for the central electrode cluster (Cz and neighboring electrodes with distance < 2) averaged across responders and nonresponders. The gray area reflects the segmented window impacted by the TMS pulse artifact. The dotted lines indicate the location of the TEP peaks (P25, N45, P70, N100, and P180). The bottom panels show the corresponding topographic distributions of the peaks averaged across responders and nonresponders. Each topography was obtained by averaging the signal in the respective TOI (P25:15–30 ms, N45: 31–55 ms, P70: 56–70 ms, N100: 71–135 ms, P180: 136–250 ms).

The single‐pulse‐evoked responses measured at T0 and at T1 of the left hemisphere are shown in the left panel in Figure 2. Pretreatment TEPs and their topographical distributions were consistent with previous studies of spTMS. Current direction had no significant influence on the presence, latency, and amplitude of peaks. The difference between T1 and T0 for the R and NR groups is shown in Figure 3. Sham stimulation between measurements revealed no significant changes and not a single cluster between measurements averaged across subjects.

#### 3.3.2 Paired‐Pulse ICF

Transcranial magnetic stimulation‐evoked potentials evoked by the paired‐pulse ICF protocol, measured at T0 and T1, are shown in Figure 2, right panel. Clusters for the P25 and P70 peaks evoked by left‐hemispheric paired‐pulse ICF stimulation were close to reaching significance; see Table S1. There were no differences between Rs and NRs. Right hemispheric stimulation with spTMS and ppTMS protocols resulted in clusters at every TOI, but no significance was reached for any cluster. Left‐hemispheric single‐pulse stimulation also resulted in nonsignificant clusters found at P25, P70, N100, and P180. No clusters were found for TOIs for right‐hemispheric stimulation.

## 4 Discussion

We investigated the effect of long‐term adjunctive AMPA‐receptor antagonist treatment on TMS‐evoked EMG and EEG potentials in adults with pharmacoresistant epilepsy. Perampanel treatment had no significant impact on TEP peak amplitudes or latencies. We found no significant modulation of TEPs and the peak TOI windows for either hemisphere or stimulation protocols. After introducing perampanel, rMT increased significantly in the R subgroup, but not in the NR subgroup.

The physiology and mechanisms underlying TMS‐evoked EEG peaks and amplitudes remain controversial [19]. Previous pharmaco‐TMS‐EEG studies have shown that single oral doses of various ASMs can modulate specific peaks in the TEP [17, 18]. We found no modulation of TMS‐evoked potentials between the pretreatment baseline and the long‐term fixed 4 mg‐dose measurement in response to spTMS or ppTMS paradigms when averaged across all subjects or groups. This contrasts with a recent study that compared two glutamate‐mediated receptor antagonists and found that perampanel reduced the P60 amplitude in the nonstimulated hemisphere in healthy subjects [20]. They speculate that this modulation may be related to interhemispheric inhibition mediated by perampanel. We could not reconfirm these findings in individuals with epilepsy starting long‐term adjunctive treatment. We also did not find significant modulation of any peaks following the ppTMS ICF protocol. No previous work utilized TMS‐EEG with ppTMS protocols to explore long‐term AMPA‐receptor antagonist peak modulation. Previous pharmacological studies with TMS‐EMG have demonstrated that the NMDA antagonist dextromethorphan and the AMPA‐type glutamate receptor antagonist memantine decreased ICF [21, 22], while the GABA_A_ agonist lorazepam also decreased ICF [27]. It is thus surprising that we do not find any peak modulation after long‐term use of perampanel for spTMS and ppTMS readouts. One explanation for the null results for spTMS and ppTMS protocols could be our limited sample size. While the Monte Carlo permutation‐based statistics we employed are relatively robust even with small samples, the statistical power to detect subtle or moderate changes in TEP peak modulation was nevertheless constrained. This limitation may have particularly affected our ability to identify smaller effect sizes that might still have clinical relevance, despite the use of appropriate statistical methods designed to maximize sensitivity in small sample analyses. Another explanation could be the rMT changes we measured. Stimulation intensity for spTMS and ppTMS is set at a fixed percentage relative to the measured rMT. Considering the significant differences in rMT within subjects, this may have normalized TEP changes. Another possibility is that, in contrast with single oral‐dose pharmaco‐EEG studies, long‐term use of medication does not modulate TEPs to a significant degree. While single‐dose studies investigating various ASMs have demonstrated modulation of specific peaks in the evoked response, long‐term use of ASMs could potentially return evoked responses to pretreatment baseline. There is little evidence at present to answer these questions as there are few longitudinal TMS‐EEG studies investigating long‐term administration of ASMs.

Resting motor threshold is thought to reflect membrane excitability and is thus affected by agents that either directly or indirectly influence the membrane potential [28, 29]. Voltage‐gated sodium channel blockers have increased rMT compared to drug‐naïve people with epilepsy and people without epilepsy. We found that people with epilepsy starting perampanel who respond to treatment have a significant increase in rMT, suggesting a reduction in cortical excitability. NRs, in contrast, had no significant change in rMT in response to adjunctive treatment. A pharmacological single‐dose study has shown similar increases in rMT after administration of perampanel in healthy subjects, suggestive of a contribution of AMPA receptors and their fast kinetics to corticospinal excitability [20]. The observed long‐term increase in rMT in Rs to perampanel treatment suggests that efficacy might be reflected in the corticospinal excitability, while those with no significant changes in seizure frequency showed no such lasting changes. The dichotomization of Rs at 50% seizure reduction is contentious. Post hoc we conducted an additional analysis examining the relationship between rMT change and seizure frequency (now expressed as seizure frequency reduction as a percentage) as continuous variables, see Figure S2. This analysis shows a significant positive correlation between the percentage change in seizure frequency reduction and the percentage change in rMT (*r* = 0.55, *p* = 0.02), suggesting that greater increases in rMT were associated with greater reductions in seizure frequency. This analysis strengthens our finding that rMT changes may serve as a potential biomarker for treatment response. Besides the demonstrated dose effect, a significant difference was observed in rMT between the two measurement centers, with SEIN requiring lower stimulator output compared to Kempenhaeghe. This difference is likely attributable to the different TMS equipment used (MagPro X100 vs. Magstim BiStim) and differences in coil diameter (14 cm vs. 9 cm), which affect the strength and focal nature of the induced electric field [30]. While this intercenter difference was statistically significant, it did not impact our primary findings because 1) our main analyses examined within‐subject changes in rMT over time, controlling for center as a fixed effect; and 2) the random intercept for subjects in our mixed‐effects model accommodated the baseline differences between participants. Nevertheless, this observation highlights the importance of standardized TMS equipment and protocols in multicenter studies and cautions against direct comparison of absolute rMT values across different TMS systems.

Our study has limitations. Firstly, concomitant medications may have potential confounding effects on rMT and ICF. Care was taken to schedule measurements at fixed times to minimize the effects of drug intake and/or daily fluctuations in cortical excitability. Blood levels of ASMs change depending on the timing of drug intake relative to the measurement time. Perampanel is prescribed to be taken before bedtime to mitigate the peak effects. Perampanel interacts with other ASMs, which may have contributed to the stability of the TEP readouts and the relatively stable response in rMT in NRs. Secondly, we did not use an auditory noise‐masking procedure during the experiments. There may have been some potential effect of the somatosensory response associated with the click generated by the coil. A recent study found that nontranscranial multisensory co‐stimulation significantly contributes to components often interpreted as the direct brain's response [19]. At the stage of off‐line analysis, we compared test TMS protocols that had nearly identical somatosensory inputs associated with the TMS clicks within subjects. In addition, between‐subject comparisons were made through the difference curve between T1 and T0. As a result, this potential confounding was limited. Lastly, the dichotomization of the participants into Rs and NRs is contentious. The < 50% reduction in seizure frequency would classify as ILAE Class 4, which is not typically regarded as a favorable outcome. Generally, complete seizure freedom is considered the optimal therapeutic goal. However, our post hoc analysis revealed a significant positive correlation between the percentage of seizure frequency reduction and the magnitude of rMT change across all participants, regardless of R classification. This relationship provides more nuanced evidence that rMT modulation tracks with clinical improvement proportionally, strengthening our finding that rMT changes may serve as a potential neurophysiological biomarker for treatment response to perampanel, even in cases where complete seizure freedom is not achieved. Lastly, we performed measurements in people with refractory epilepsy, thus severely limiting the chance of seizure freedom after starting adjunctive administration with perampanel.

## 5 Concluding Statements and Future Perspective

We demonstrated that the long‐term effects of perampanel treatment in people with epilepsy do not lead to significant modulation of any of the TEP peaks. This contrasts with the more basic EMG rMT measure, which significantly reduced corticospinal excitability in the R subgroup. In individual cases, changes in rMT may be monitored or used as a promising biomarker to evaluate lasting changes in overall (motor) cortical excitability, treatment adjustments, and outcome. Future research should be focused on exploring the effect of long‐term use of ASMs and the effect on TMS‐EMG/EEG measures as potential biomarkers for treatment outcome.

## Author Contributions

R.M.H., J.P.D., P.R.B., M.Z., J.W.S., and G.H.V. contributed to the study's conception; all authors contributed to its design; R.M.H., and J.P.D. contributed to acquiring the data; R.M.H. prepared the figures; and all authors contributed to analyzing the data and editing.

## Conflicts of Interest

R.D.T. reports lecture and consultancy fees from Medtronic, UCB, Theravarance, Zogenix, Novartis, and Arvelle and grants from EpilepsieNL, Medtronic, Michael J Fox Foundation, NewLife Wearables, and the Netherlands Organisation for Health Research and Development (843002707). P.R.B. receives lecture fees from NovoCure and Aurikamed. J.W.S. reports personal fees from Eisai, UCB Pharma, and Angelini Pharma and grants from Angelini and UCB to his department outside the submitted work. G.H.V. reports grants from the Dutch National Epilepsy Fund and Eisai. All other authors have no disclosures to make.

## Supporting information


Data S1.

## Data Availability

The data that support the findings of this study are not publicly available due to restrictions related to participant consent. Specifically, consent for public sharing of the data was not obtained at the time of collection. However, the data are available from the corresponding author upon reasonable request.
