# Supplementary material for: Cortical Excitability Before and After Long‐Term Perampanel Treatment for Epilepsy
Source: Ann Clin Transl Neurol. 2025 Apr 17;12(6):1256–64. doi: 10.1002/acn3.70044 (PMC12172129; doi:10.1002/acn3.70044)
Supplement: Supplementary file 1 — Data S1. [file ACN3-12-1256-s001.docx]

Supplementary information


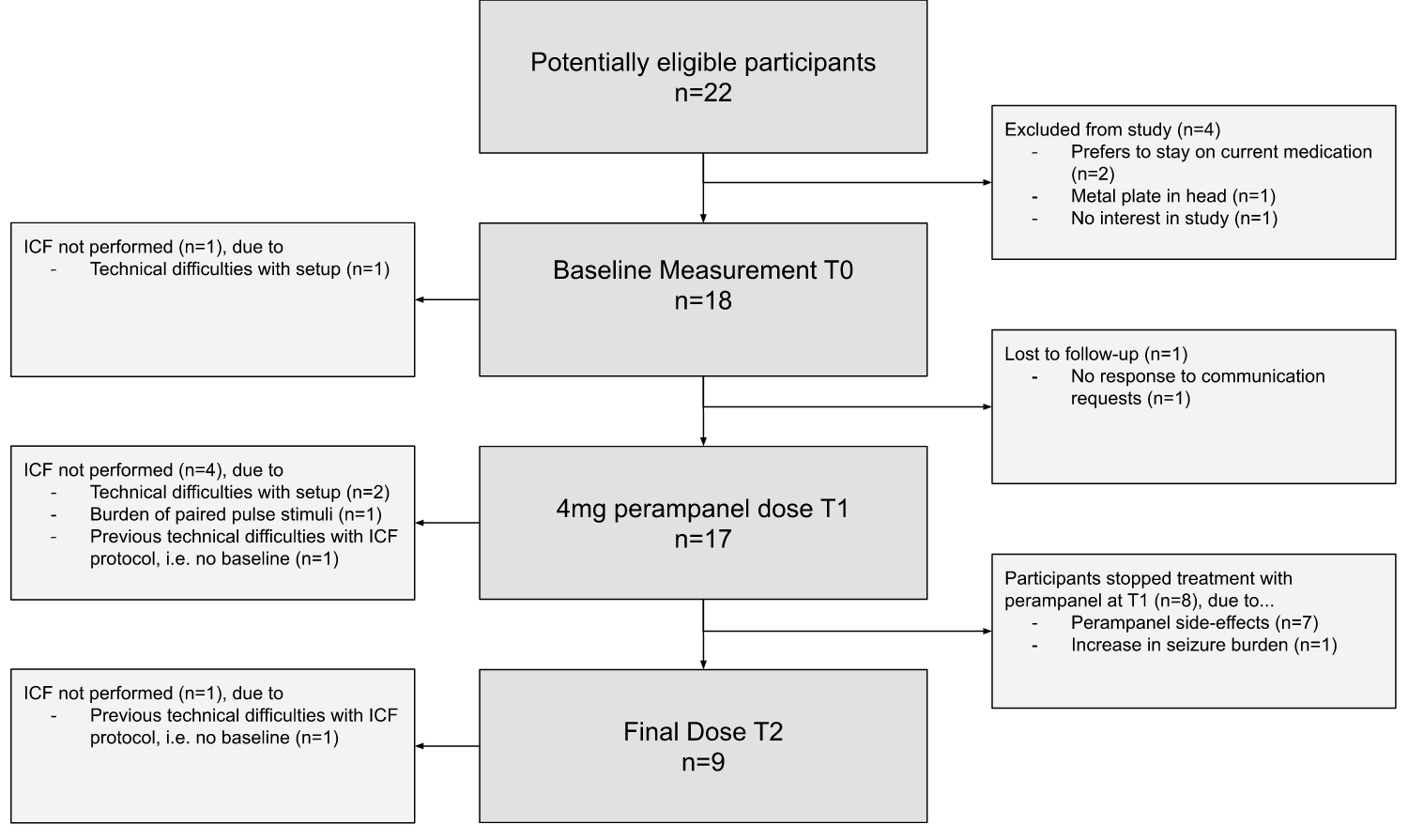


**Fig. S1.** Study flow-chart.


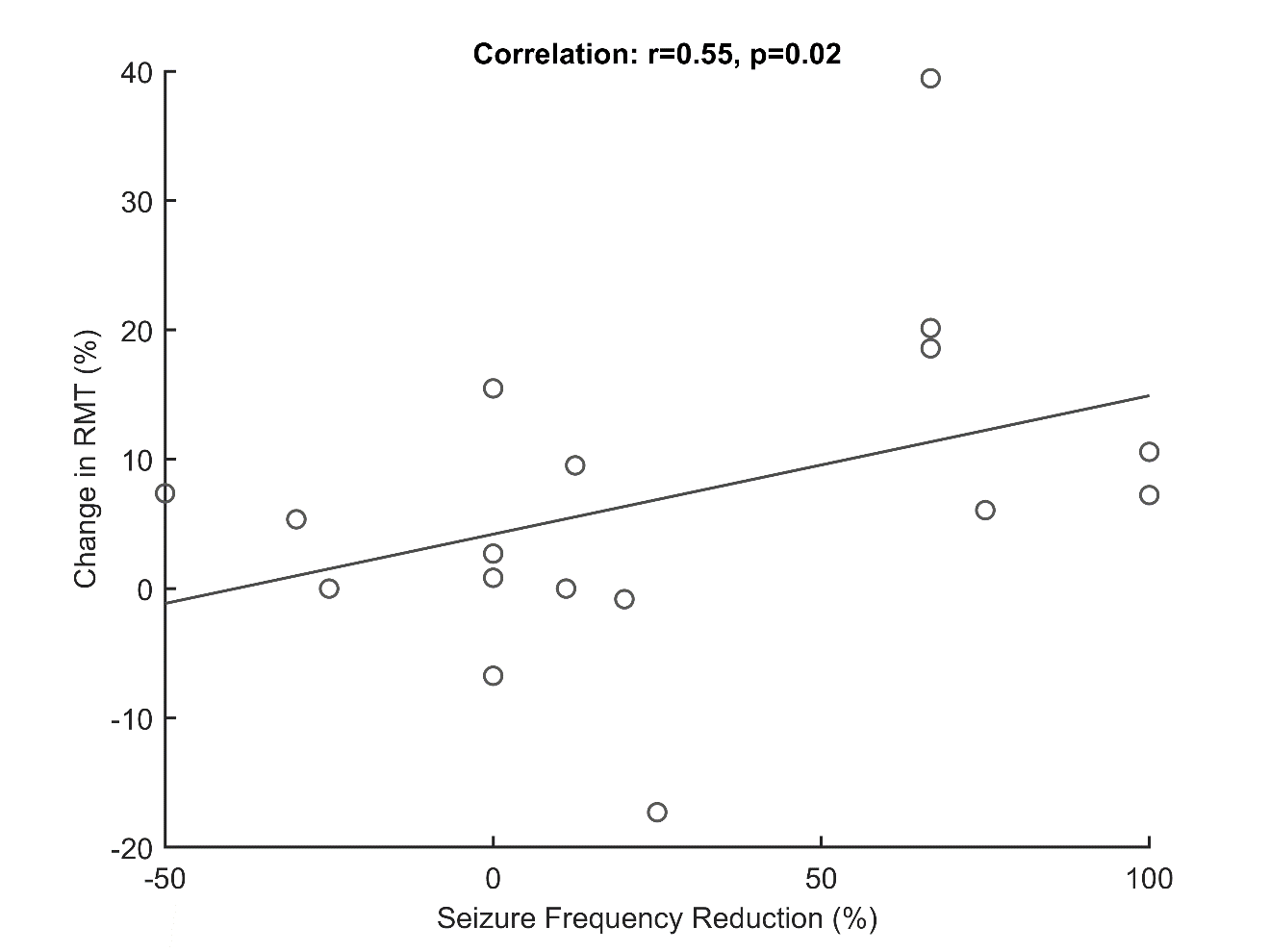


**Fig. S2.** Correlation analysis of the seizure frequency reduction (%) relative to the change in mean rMT (%).

***Table S1. Cluster statistics for TMS-evoked EEG peaks for the clockwise versus counterclockwise current direction at baseline.***

|  |  | **P25** | | | **N45** | | | **P70** | | | **N100** | | | **P180** | | |
| --- | --- | --- | --- | --- | --- | --- | --- | --- | --- | --- | --- | --- | --- | --- | --- | --- |
| **Prot** | | **n** | **p** | **n** | | **p** | **n** | | **p** | **n** | | **p** | **n** | | **p** |  |
| SP | | - / - | - / - | - / - | | - / - | - / - | | - / - | - / - | | - / - | - / - | | - / - |  |
| ICF | | 1  / 1 | 0.44  / 0.47 | - / - | | - / - | - / - | | - / - | - / - | | - / - | - / - | | - / - |  |

*For each comparison we show the number of found positive and/or negative clusters (n of 1 / 2 means a single positive and two negative clusters were found) and corresponding p-value statistics for the cluster with highest summed T-values. Sham results are not shown because not a single cluster was found.*

***Table S2. Cluster statistics for TMS-evoked EEG peaks for the measurement at 4mg perampanel (T1) in comparison to baseline (T0).***

|  |  | **P25** |  | **N45** |  | **P70** |  | **N100** |  | **P180** |  |
| --- | --- | --- | --- | --- | --- | --- | --- | --- | --- | --- | --- |
|  |  |  |  |  |  |  |  |  |  |  |  |
| **Prot** | **Hemi** | **n** | **p** | **n** | **p** | **n** | **p** | **n** | **p** | **n** | **p** |
| SP | Right | - / - | - / - | - / - | - / - | - / - | - / - | - / 1 | - / 0.213 | 1  /  3 | 0.329 / 0.304 |
|  | Left | - / - | - / - | - / - | - / - | - / - | - / - | - / - | - / - | 2 /  4 | 0.205 / 0.533 |
| ICF | Right | - / - | - / - | - / - | - / - | - / - | - / - | - / - | - / - | 1  / 2 | 0.320 / 0.410 |
|  | Left | 1  / - | 0.089 / - | - / - | - / - | 2  / - | 0.064 / - | 1 / 1 | 0.226 / 0.412 | - / 2 | - / 0.284 |

*For each comparison we show the number of found positive and/or negative clusters (n of 1 / 2 means a single positive and two negative clusters were found) and corresponding p-value statistics for the cluster with highest summed T-values. Sham results are not shown because not a single cluster was found.*

***Table S3. Cluster statistics for TMS-evoked EEG peaks for the responders non-responder comparison.***

|  |  | **P25** |  | **N45** |  | **P70** |  | **N100** |  | **P180** |  |
| --- | --- | --- | --- | --- | --- | --- | --- | --- | --- | --- | --- |
| **Prot** | **Hemi** | **n** | **p** | **n** | **p** | **n** | **p** |  | **n** | **p** | **n** |
| SP | Right | 1  /  2 | 0.112  /  0.119 | - /  1 | - / 0.130 | 1  /  1 | 0.118  /  0.368 | 1  / - | 0.295  / - | 2  /  4 | 0.511 / 0.630 |
|  | Left | 1  / - | 0.432  / - | - / - | - / - | 1  / - | 0.151  / - | - /  1 | - / 0.491 | 2  /  6 | 0.304 / 0.324 |
| ICF | Right | 1  / - | 0.226  / - | 1  / - | 0.089  / - | 2  / - | 0.146  / - | 1  /  1 | 0.412  /  0.402 | 1  /  1 | 0.268 / 0.123 |
|  | Left | - / - | - / - | - / - | - / - | - / - | - / - | - /  1 | - / 0.266 | 1 /  1 | 0.177 / 0.076 |

For each comparison we show the number of found positive and/or negative clusters (n of 1 / 2 means a single positive and two negative clusters were found) and corresponding p-value statistics for the cluster with highest summed T-values. Sham results are not shown because not a single cluster was found.
